# Extracellular matrix physical properties regulate cancer cell morphological transitions in 3D hydrogel microtissues

**DOI:** 10.1016/j.actbio.2025.12.008

**Published:** 2025-12-04

**Authors:** Ayda Pourmostafa, Gabrielle Uskach, Mohammad Jafari, Elvan Dogan, Swaprakash Yogeshwaran, Teresa L. Wood, Sobhan Ghaeini-Hesaroueiye, Lin Han, Farid Alisafaei, Amir K. Miri

**Affiliations:** a Department of Biomedical Engineering, New Jersey Institute of Technology, Newark, NJ, USA; b Department of Mechanical and Industrial Engineering, New Jersey Institute of Technology, Newark, NJ, USA; c Department of Pharmacology, Physiology, and Neuroscience, New Jersey Medical School, Rutgers University, Newark, NJ, USA; d School of Biomedical Engineering, Science and Health Systems, Drexel University, Philadelphia, PA, USA

**Keywords:** Remodeling, Cancer mechanobiology, Gelatin matrix, Minimum free energy

## Abstract

Solid tumor cells can adopt a range of morphological states linked to distinct functional behaviors during tumor progression. Some remain in a proliferative state, forming tight clusters, others detach and elongate into an invasive state, and some retain a rounded amoeboid form with minimal matrix adhesion. However, factors determining which morphological state a cell adopts remain poorly understood. We used a combined theoretical and experimental framework to study how extracellular matrix (ECM) mechanics regulate solid tumor cell morphology in three-dimensional (3D) environments. We developed a theoretical mechanical energy model based on the minimum energy principle, which suggests that a cell will adopt the morphological state (rounded, elongated, or clustered) that minimizes the total energy of the cell-ECM system. Using MDA-MB-231 breast cancer cells, we established a reliable protocol for encapsulating cells into 3D naturally-derived hydrogels with controlled stiffness. We confirmed the model’s results *in vitro* over an extended culture period. In soft ECMs, cells transitioned over time to an elongated morphology, while in stiff ECMs, cells favored clustered configurations. These transitions were governed by the hydrogel-based ECM’s physical, not chemical, properties, as confirmed using chemically distinct yet mechanically matched composite matrices. These new insights have implications for solid tumor cell invasion modeling *in vitro*.

## Statement of significance

We study the fundamental question of how solid tumor cells adapt their morphology in response to the physical characteristics of the extracellular matrix. This work establishes a robust experimental platform for studying cellular markers in triple-negative breast cancer (TNBC) cells, followed by a biophysical modeling of the cell invasion. Cell clustering was observed in stiffe ECMs, while an elongated morphology was observed in soft ECMs. Our theoretical modeling revealed how the biophysical properties of the matrix can impact cell morphology and invasion behavior. This work can contribute to personalized medicine by making more effective, tailored cancer models.

## 1. Introduction

The extracellular matrix (ECM) provides structural and mechanical cues that regulate solid tumor cell behavior [1–4]. The mechanical properties of the ECM, such as stiffness and pore architecture, play crucial roles in determining how cells interact with their microenvironment [5–9]. The mechanical properties of the native tissue can vary widely by location within the body [10,11]. In the case of tumors, ECM remodeling leads to a wide range of mechanical properties, from soft, porous regions to dense, fibrotic areas [12–14]. These mechanical variations influence cell adhesion, migration, and cytoskeletal organization, ultimately shaping cancer progression [15,16]. Increased ECM stiffness, often driven by collagen deposition, is associated with tumor aggressiveness and poor patient outcomes [17,18]. However, how cells interpret and respond to these mechanical signals in three-dimensional (3D) microenvironments remains an open question.

In traditional 2D environments, substrate stiffness is the dominant factor governing cell morphology [19]. On a soft surface, cells often retain a rounded morphology with minimal adhesion to the substrate, while on stiffer surfaces, they spread and generate higher contractile forces [15,20]. However, in 3D ECMs, cells exhibit fundamentally different behaviors [21–23]. Unlike in 2D, where stiffness alone dictates morphology, a synergistic combination of stiffness and pore size influences how cells respond to their surroundings in 3D ECMs [9]. Pore size affects cell confinement, migration modes, and access to biochemical signals, making it a critical but often overlooked parameter in engineered 3D ECMs [24–26]. This added complexity in 3D raises the fundamental question of how cells adapt their morphology when exposed to different combinations of ECM stiffness and pore size.

Understanding how cancer cells adapt their morphology within 3D microenvironments is essential for elucidating the mechanisms of tumor invasion and engineering tunable tumor models. In patient tumors, the mechanical properties of the ECM are highly heterogeneous, with regions of both soft and stiff tissue coexisting due to fibrosis, remodeling, and variations in stromal composition [27,28]. This heterogeneity may lead cells to adopt one of three distinct morphological states: (i) a “rounded” morphology, in which the cell remains spherical and migrates through matrix pores with minimal adhesion (amoeboid mode of migration); (ii) an “elongated” morphology, characterized by cell spreading and the extension of protrusions to engage with the matrix (mesenchymal mode of migration); or (iii) a “clustered” morphology, where the cell proliferates and forms small interconnected groups [29–32]. Each of these morphological states may have distinct implications for cell invasion and disease progression, including differences in migration potential, resistance to mechanical stress, and interaction with the tumor microenvironment [33–35]. A quantitative framework linking ECM mechanical properties to cell morphology in 3D remains lacking in the field.

To study this phenomenon *in vitro*, we used a combination of theoretical and experimental frameworks for triple-negative breast cancer (TNBC). Our theoretical model is based on the minimum energy principle, [36–40]. which posits that a cell will adopt the configuration that minimizes the total energy of the cell-ECM system [41–44]. We assessed these results experimentally by culturing individual MDA-MB-231 cells within 3D ECMs with varying mechanical properties. MDA-MB-231 is a well-established model for triple-negative breast cancer, known for its invasive, mesenchymal-like phenotype and mechanical sensitivity to matrix cues, making it particularly suitable for investigating morphology transitions in response to ECM mechanics [45]. We used hydrogel engineering and biomaterials science to optimize the biofabrication protocol for reliable 3D tumor models for up to three weeks. The novelty of this study lies in combining theoretical modeling with long-term 3D culture to reveal dynamic morphology transitions of invasive cancer cells. Our findings support the emerging concept that cell morphology follows mechanical rules, adopting configurations that minimize the energy of the cell-ECM system. This predictive framework provides new insights into how ECM mechanics regulate tumor cell behavior in complex microenvironments. By integrating theoretical modeling and experimentation, our study provides new insights into the biophysical mechanisms that govern cancer cell morphology in complex microenvironments. Such insights could guide the design and development of tunable *in vitro* models to screen anti-cancer drugs that target the physical tumor microenvironment to limit cancer invasion.

## 2. Materials and methods

### 2.1. Materials

Cell culture reagents included Dulbecco’s Modified Eagle Medium (DMEM; Corning, USA), fetal bovine serum (FBS; Corning, USA), Penicillin-Streptomycin (10,000 U/mL; Gibco, Thermo Fisher Scientific, Cat. 15,140,122), and Dulbecco’s phosphate-buffered saline (DPBS; Sigma-Aldrich). For hydrogel synthesis, porcine skin gelatin (CAS 9000–70–8), methacrylic anhydride (CAS 760–93–0), N, N-dimethylformamide (DMF), sodium hydroxide (NaOH), ethanol, dialysis tubing (12–14 kDa cutoff; Spectrum Labs), and lithium phenyl-2,4,6-trimethylbenzoylphosphinate (LAP) were used. Hyaluronic acid (BioSynthesis Inc., Germany) was also incorporated for composite hydrogel preparation. The human breast cancer cell line MDA-MB-231 was obtained from ATCC (USA). For immunostaining, formaldehyde (Molecular Biology, 36.5–38 % in H_2_O, Sigma-Aldrich), Triton X-100 (Thermo Scientific Chemicals, CAS: 9002–93–1), bovine serum albumin (BSA; Thermo Scientific, USA), Phalloidin, Alexa Fluor^™^ 488 conjugate (Thermo Fisher, Cat. A12379), and DAPI (Sigma-Aldrich, USA) were used. Cell viability and metabolic activity were assessed with the LIVE/DEAD^™^ Viability/Cytotoxicity Kit (Invitrogen, USA) and the CCK-8 assay (Sigma-Aldrich). For cytoskeletal inhibition studies, cytochalasin D (Sigma-Aldrich, C2618) and blebbistatin (Sigma-Aldrich-B0560) were applied.

### 2.2. Cell preparation

A human breast cancer cell line (MDA-MB-231, ATCC; Manassas, VA) was chosen based on its adaptability in response to the matrix. Cells were cultured in Dulbecco’s Modified Eagle Medium mixed with 10 % v/v fetal bovine serum (FBS) and 1 % v/v Penicillin-Streptomycin, following standard practices.

### 2.3. Material preparation

GelMA was synthesized according to an established protocol [46]. In a 100 mL glass flask, Dulbecco’s phosphate-buffered saline (DPBS) (Sigma-Aldrich, USA) was mixed with 10 % w/v porcine skin gelatin (CAS Number 9000–70–8; Sigma-Aldrich). The flask was covered to prevent evaporation and stirred using a magnetic stir bar on a hot plate at 60 °C until fully dissolved (approximately one hour). After the gelatin was completely dissolved in the DPBS, 5 mL of methacrylic anhydride (CAS: 760–93–0, Sigma-Aldrich) was slowly pipetted into the solution. The temperature was then lowered to 50 °C, and the solution was stirred and allowed to react for one hour. To stop the reaction, pre-warmed DPBS, five times the volume of the initial solution, was added. GelMA was dialyzed using dialysis tubing (12–14 kDa molecular weight cut-off) for one week to remove excess methacrylic anhydride.

Hyaluronic acid (BioSynthesis Inc., Germany) was dissolved in PBS (2 g in 100 mL) and stirred at 4 °C until completely dissolved. Then, 67 mL of N, N-dimethylformamide (DMF, Sigma) was added to the hyaluronic acid solution and stirred until well mixed. An appropriate amount of methacrylic anhydride (CAS: 760–93–0, Sigma-Aldrich, USA) was added dropwise, and 1 M sodium hydroxide (NaOH, Sigma) was used to adjust the pH. The solution was reacted at 4 °C for 24 h, then mixed with 95 % ethanol to precipitate and crystallize the product. The precipitate was washed with ethanol and deionized water and subsequently dissolved in deionized water. The solution was dialyzed using 12–14 kDa dialysis tubing for one week. The NMR spectrum was then used to confirm the efficiency of chemical yield and the degree of acrylation. After freeze-drying, a hyaluronic acid methacrylate (HAMA) stock solution (2 % w/v) was prepared in DPBS and pre-warmed to 50 °C with constant stirring. Hydrogel precursors were prepared by dissolving stock solutions and stirring at 50 °C. Stiff formulations contained 7 % GelMA (Stiff GelMA) or 3 % GelMA + 1 % HAMA (Stiff HAMA-GelMA), while soft formulations consisted of 5.7 % GelMA (Soft GelMA) or 2 % GelMA + 0.7 % HAMA (Soft HAMA-GelMA). Lithium phenyl-2,4,6-trimethylbenzoylphosphinate (LAP, 1 % w/v) was added as the photo-initiator, and all solutions were filtered through 0.22 μm syringe filters.

### 2.4. Bioink preparation and biofabrication

GelMA and HAMA-GelMA solutions were mixed with pelleted cells at the desired concentration (1 × 10^6^ cells/mL) to form cellular hydrogels. Acellular hydrogels were prepared in the same way, without the addition of cells. Using our extrusion bioprinter, we printed predefined models and then crosslinked the constructs using post-extrusion light exposure [26]. Disk-shaped samples (8 mm in diameter and 2.5 mm in height) were fabricated in triplicate for each time point (Days 0, 1, 3, 7, 14, and 21). Samples were crosslinked under UV light for 3 min at an intensity of 3800 × 100 μJ/cm^2^ (VWR). After crosslinking, cellular samples were incubated in DMEM supplemented with 10 % v/v fetal bovine serum (FBS) and 1 % v/v Penicillin-Streptomycin (Pen/Strep) (Corning, USA), while acellular samples were incubated in DPBS. All samples were maintained in anti-adherent-coated using anti-adherence Rinsing Solution, AggreWell (Stemcell Technologies, USA) 12-well plates.

### 2.5. Mechanical characterization

Standard compression testing was performed to measure the stiffness under compressive load. Samples were placed on a metal flat plate of the universal testing machine (Instron, MA, USA), and the tests were performed using a strain rate of 0.5 mm/min. Regression was used to determine the linear area, and Young’s modulus was calculated as the slope of the stress-strain curve from 0–10 % strain.

Atomic force microscopy (AFM)-based nanoindentation was applied to quantify the effective modulus of both soft and stiff hydrogels at the microscale, following our established procedure [47]. In brief, nanoindentation was applied to the surfaces of freshly made hydrogels using polystyrene microspherical tips (*R* ≈ 12.45 μm, nominal *k* ≈ 0.03 N/m, HQ:CSC38/tipless/No Al, cantilever B, NanoAndMore) and a Dimension Icon AFM (Bruker Nano) at 10 μm/s indentation rate up to ≈ 2 μm maximum indentation depth in 1 × PBS. For each hydrogel group, indentation was repeated on at least *n* = 3 samples, with at least 12 randomly chosen locations tested on each sample to account for heterogeneity. The effective modulus, E_ind_, was calculated by fitting the entire portion of each force-distance loading curve to the Hertz model, assuming a Poisson’s ratio of 0.49 for highly swollen hydrogels [48].

### 2.6. Mass characterization

After mechanical characterization was completed, samples were weighed with a balance scale. Samples were fixed in 4 % paraformaldehyde (Sigma-Aldrich) for 2 h. Paraformaldehyde was removed, and the samples were kept at 4 °C in DPBS. Samples were frozen at −80 °C for 24 h and lyophilized for 48 h. Mass residuals were calculated using the mass of the chosen time point ($M_{x}$) divided by the average mass of day 0 samples ($M_{0}$):

$$Mass\;Residuals(\%)=\left[M_{x}/M_{0}\right]\times 100$$

### 2.7. Swelling measurement

Fixed samples were weighed again and placed in centrifuge tubes with perforated caps to allow airflow. The samples were then freeze-dried to produce dry scaffolds. These dry scaffolds were weighed using a balance scale. The swelling ratio was calculated using the following formula, where $M_{w}$ is the mass of the wet sample and $M_{d}$ is the mass of the corresponding dry sample [26,49]:

$$Swelling\;Percentage(\%)=\left[\left(M_{w}-M_{d}\right)/M_{w}\right]\times 100$$

### 2.8. Pore size measurement

Hydrogel precursor solutions were cast in PDMS molds to form disk-shaped samples (8 mm × 5 mm) and equilibrated in culture medium at 37 °C for 24 h. Samples were rinsed twice with 1 × PBS and fixed with 4 % formaldehyde for 1 h at room temperature. Fixed hydrogels were dehydrated through a graded ethanol series (DI water, 30 %, 50 %, 70 %; 5 min each, 2–3 cycles per step) and lyophilized. The dried samples were sectioned, sputter-coated with platinum (EMITECH, K575X), and imaged using a field-emission scanning electron microscope (JSM-7900F, JEOL). Pore size was determined by measuring five diameters per pore at different angles, and the mean value was reported as the pore diameter using ImageJ software, as shown in Fig. S1.

### 2.9. Static diffusion

Fixed hydrogels were rewashed with DI water three times for 20 min each, then cut into smaller pieces and weighed. The hydrogels were immersed in Rhodamine B (RhB) at a concentration of 0.5 mg/mL for 3 min, after which they were removed, washed twice with DI water to eliminate surface-bound RhB, and placed into a 24-well plate or separate beakers containing 1 mL of DI water overnight. The following day, the samples were measured at a fluorescent excitation peak at 546 nm and an emission peak at 567 nm. The gel samples were then immersed in a RhB bath (0.5 mg/mL) for 2 h to reach a steady state, and the release measurement step was repeated. We assumed 1-D diffusion transport and used Fick’s law to calculate the diffusion coefficient [50].

### 2.10. Cell viability and metabolic activity

Cellular responses to GelMA and HAMA-GelMA hydrogels were evaluated using Live/Dead staining (Invitrogen^™^ LIVE/DEAD^™^ Viability/Cytotoxicity Kit) and the CCK8 assay (Sigma-Aldrich). A Nikon ECLIPSE Ti2 microscope was used for imaging. ImageJ software was employed to quantify live and dead cells and calculate cell viability as a percentage. Cell metabolic activity was quantified using the CCK-8 assay (Sigma-Aldrich) according to the manufacturer’s instructions. After incubation, absorbance was measured at 450 nm using an Agilent BioTek Synergy H1 multimode reader, and results were normalized to negative controls. Data were expressed as optical density (OD) values representing the relative metabolic activity of encapsulated cells.

### 2.11. Myosin II and actin polymerization inhibition

Inhibition of actomyosin-dependent contractility was achieved by pretreating resuspended MDA-MB-231 cells with DMEM containing cytochalasin D (Sigma-Aldrich, St. Louis) at a concentration of 2 μM, or blebbistatin (Sigma-Aldrich, St. Louis) at 10 μM for 30 min before making the hydrogel matrix at 37 °C with 5 % CO2. This ensured that experiments captured the effect of the inhibitors at the earliest stage of matrix remodeling. After the first hour of incubation, each model was filled with DMEM containing the chemicals as mentioned above, and the media was changed every day to maintain the effect of the chemicals on the cells.

### 2.12. Cell morphological analysis

The samples were fixed in 4 % (v/v) paraformaldehyde and washed three times with 1 × PBS. They were then permeabilized using 0.1 % Triton X-100 (Sigma-Aldrich) in DPBS for 15 min, followed by blocking with 1 % bovine serum albumin (BSA) blocking buffer for 1 hour. Samples were subsequently incubated overnight at 4 °C with 1:100 FITC-conjugated phalloidin (1 μg/mL, Invitrogen, Thermofisher) in 1 % BSA overnight at 4 °C. Hydrogels were counterstained with DAPI (0.1 μg/mL, Sigma Aldrich, USA) for 15 min and imaged with a Nikon ECLIPSE Ti2 microscope.

Circular-shaped hydrogels were analyzed in both the center and edge regions, with the edge region defined as the area within 2 mm of the hydrogel perimeter and the center region defined as the area within a 2 mm radius from the geometric center. Quantification of immunofluorescent staining intensity was performed using ImageJ, where the mean pixel intensity was normalized to the number of nuclei in each field of view. Spheroid size, distribution, and roundness were evaluated from three representative images per group and time point. Cell circularity was quantified in ImageJ using the built-in “Circularity” shape descriptor.

$$Circularity=4\pi (Area)/{\left(\mathrm{Perimeter}\right)}^{2}$$

Cell fluorescence staining was performed using independent biological replicates and repeated three times. For quantitative image analysis, three replicate samples per condition were analyzed, and (*n* ≥ 10 independent cells/clusters per sample were quantified using ImageJ (NIH, USA).

### 2.13. Statistics and reproducibility

All statistical analyses were performed using *GraphPad Prism* (10.3.1). Data are presented as mean ± standard error of the mean (s.e.m.) unless otherwise stated. The number of independent biological replicates (*n* = 3) refers to experiments conducted with cells from different passages or independent hydrogel matrix sample preparations. Variance between groups was analyzed using one-way or two-way analysis of variance (ANOVA), depending on the number of experimental factors, followed by appropriate post hoc multiple-comparison Tukey’s tests to identify statistically significant differences between conditions. Normality and homogeneity of variance were confirmed prior to applying parametric tests. Statistical significance was defined as follows: 0.01 *<* *p ≤ 0.05, **p ≤ 0.01, ***p ≤ 0.001, and ****p ≤ 0.0001. All experiments were repeated independently to ensure reproducibility and consistent trends across replicates.

### 2.14. Theoretical modeling

A theoretical model was developed to estimate the total energy of the cells and ECM. We developed a 3D continuum-based constitutive model in which the total cytoskeletal response arises from the coupled contributions of (i) myosin molecular motors, (ii) actin filaments, and (iii) microtubules, as illustrated schematically in Fig. 2B. This constitutive model was implemented into a finite element framework, such that each element follows the same underlying material law. The finite element simulations were performed by implementing the theoretical model into a UMAT subroutine within Abaqus. This framework was used to compute the cell-ECM energy, as described in Supplementary Information, across a range of ECM stiffnesses and morphological configurations. Specifically, we modeled three scenarios: a single round cell, a spherical cluster, and a single elongated cell, embedded within a soft or stiff ECM. For each case, we calculated the individual contributions of motor-work, chemical, and mechanical energy within the cell, along with the strain energy stored in the ECM due to matrix deformation. All energy terms were evaluated as volumetric densities, as described in the Supplementary Information. The total energy associated with each component was then obtained by multiplying its volumetric density by the corresponding cell or ECM volume for each scenario. These simulations allowed us to quantify how total energy varies with ECM stiffness and cell morphology.

#### Single round cell:

The cell was modeled as a sphere with a diameter of 25 μm (Fig. S6), embedded at the center of an ECM domain. The ECM was represented as a cube with a side length of 800 μm, sufficiently large to eliminate any boundary effects on the mechanical response of the cell. In this scenario, the cell does not expand; therefore, the ECM energy was computed solely based on the strain energy generated by cell contraction. A fine mesh density was selected to ensure sufficient resolution for capturing deformation gradients and achieving numerical accuracy throughout the domain. The cell domain was discretized using 68,616 linear tetrahedral elements (C3D4), while the ECM was meshed with a combination of 8432 C3D4 elements near the cell and 63,992 linear hexahedral elements (C3D8) in regions distant from the cell.

#### Spherical cluster:

The spherical cluster configuration was modeled as an aggregate of six individual cells (Fig. S6), each represented as a sphere with a diameter of 25 μm, arranged symmetrically with marginal overlap to form a compact structure. This cluster was embedded at the center of an ECM domain modeled as a cube with a side length of 800 μm. To compute the energy stored in the ECM due to cluster expansion, a separate simulation was conducted in which the ECM contained a central void equal in size to a single cell. A displacement field was then applied to the void boundary to expand it to the final size of the six-cell aggregate, allowing estimation of the ECM strain energy associated with cell cluster expansion. After calculating the ECM energy resulting from matrix deformation due to cluster expansion driven by cell proliferation, we next calculated the ECM energy associated with cluster contraction. The ECM energy contribution due to cell contraction was determined from a coupled simulation containing both the cluster and the surrounding ECM. The cluster was discretized with 222,545 linear tetrahedral elements (C3D4). The ECM mesh included 39,415 C3D4 elements near the cluster and 62,250 linear hexahedral elements (C3D8) in the outer region.

#### Elongated cell:

The elongated cell configuration was modeled as a long prolate spheroid (Fig. S6), with a minor axis of 25 μm and a major axis of 150 μm, positioned at the center of a surrounding ECM domain. The ECM was again defined as a cube with a side length of 800 μm, consistent with other simulation setups. To isolate the energy contribution from matrix deformation caused by cell elongation, a separate simulation was performed in which the ECM initially included a central void corresponding to a spherical cell. A prescribed displacement was then applied to the void boundary to expand it to the shape and dimensions of the elongated cell, enabling the calculation of the ECM strain energy resulting from cell expansion. The additional ECM energy due to cell contractility was computed in a separate coupled model, including both the elongated cell and the ECM. Note that the processes of cell spreading (elongation) and cell contraction occur cyclically over time, driving the transition from a small, round morphology to a fully spread and elongated state, as we have recently demonstrated [39]. However, for simplicity, we simulated each of these two processes separately and calculated the energy associated with each individually. The cell geometry was meshed using 211,039 linear tetrahedral elements (C3D4). The ECM mesh consisted of 32,041 C3D4 elements in proximity of the cell and 67,096 linear hexahedral elements (C3D8) farther from the cell to ensure accurate resolution of localized and bulk matrix deformation.

## 3. Results

### 3.1. Engineered 3D hydrogels as a model for tumor ECM mechanics

**Fig. 1.**
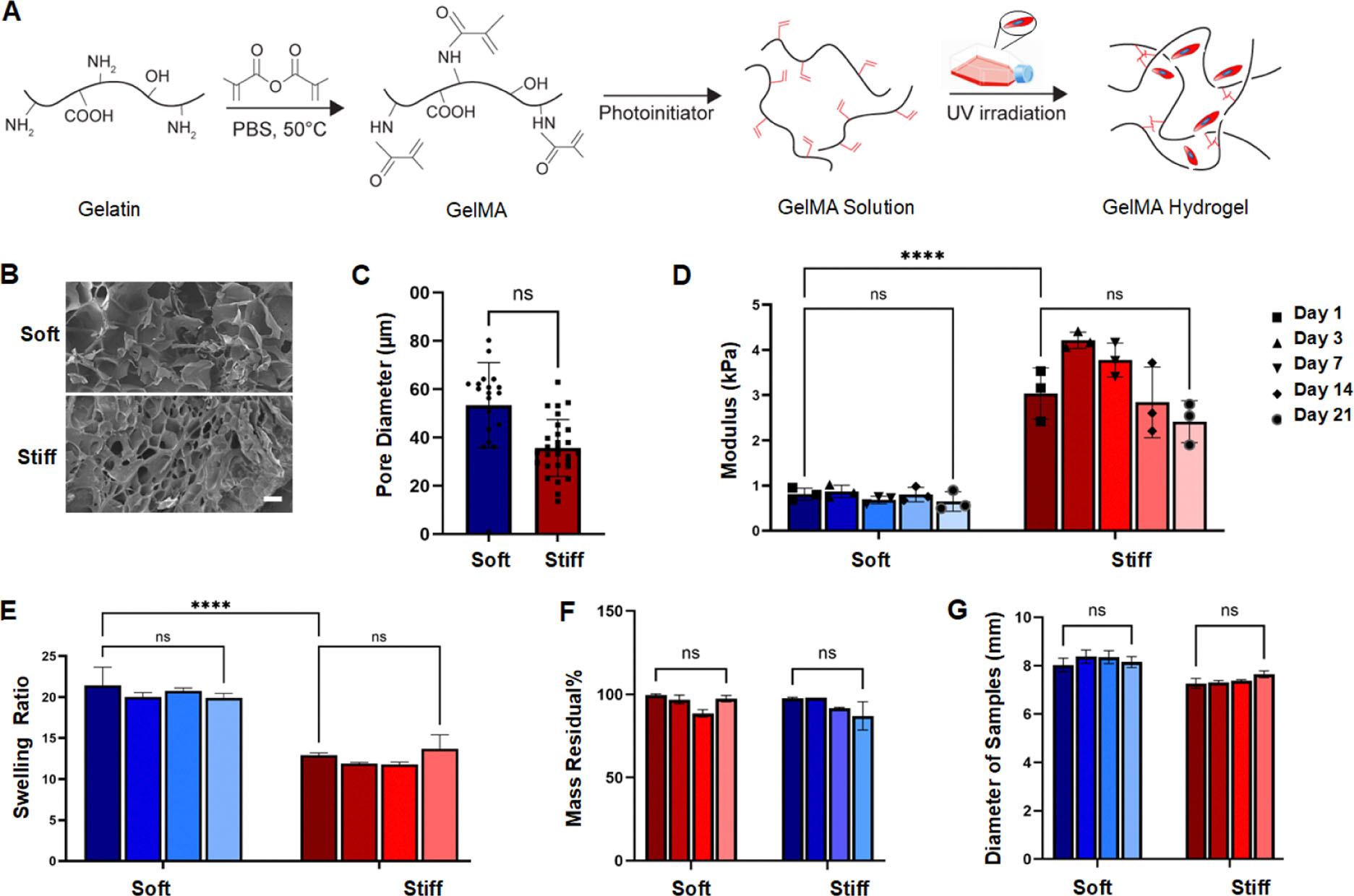
Engineering and characterization of 3D GelMA hydrogels as a stable platform for cell culture. (A) Schematic representation of hydrogel preparation and cell encapsulation. (B) Scanning electron microscopy (SEM) images of soft and stiff GelMA hydrogels (scale bar, 100 μm). (C) Quantification of pore size for soft and stiff hydrogels (*n* = 3 SEM images of each condition). (D) Stiffness measurements of cell-laden hydrogels over time (*n* = 3). (E–G) Characterization of cell-laden hydrogels over 14 days, showing constant (E) swelling ratio, (F) dry mass residual percentage, and (G) hydrogel sample size, confirming system stability. ns *p >* 0.05, *p ≤ 0.05, **p ≤ 0.01, ***p ≤ 0.001, ****p ≤ 0.0001. Bar heights represent mean values, and error bars represent ± standard error of the mean (*n* = 4). Data have been collected from 3 different biological repeats.

The mechanical properties of the ECM play a crucial role in shaping cell behavior in solid tumors, where regions of varying stiffness and pore sizes coexist due to differences in ECM density and structural remodeling. To replicate these conditions, we engineered 3D gelatin methacryloyl (GelMA) hydrogels with two distinct bulk mechanical properties: a soft ECM (~ 1 kPa) with large pores (50–100 μm) and a stiff ECM (~ 3.5 kPa) with smaller pores (10–50 μm) (Fig. 1A–D) [50,51]. GelMA is derived from gelatin, a denatured form of collagen, replicating native major ECM cues for tumor cells [52]. These properties mimic the range of mechanical conditions found *in vivo*, where increased collagen concentration leads to simultaneous stiffening and pore size reduction [17,32,53].

To assess how the mechanics of hydrogel-based ECM influence cell morphology, we encapsulated MDA-MB-231 cells within both soft and stiff ECMs (Fig. S2–5). In both conditions, we used a cell density of 1 × 10^6^ cells/mL [54]. To ensure that the hydrogels remained structurally and chemically stable over time, we monitored swelling ratio (Fig. 1E), mass residual (Fig. 1F), and tissue diameter (Fig. 1G) over 14 days. All three parameters remained almost constant, indicating that the hydrogel structure was preserved throughout the experiment. In particular, the mass residual remained unchanged, suggesting minimal degradation and confirming that matrix metalloproteinase (MMP) activity did not significantly alter the ECM. These results demonstrate that our hydrogel system provides a stable and physiologically relevant platform to study how ECM mechanics regulate cell morphology, as suggested by our previous study [26].

### 3.2. Theoretical modeling: cells are elongated in soft ECM and clustered in stiff ECM

**Fig. 2.**
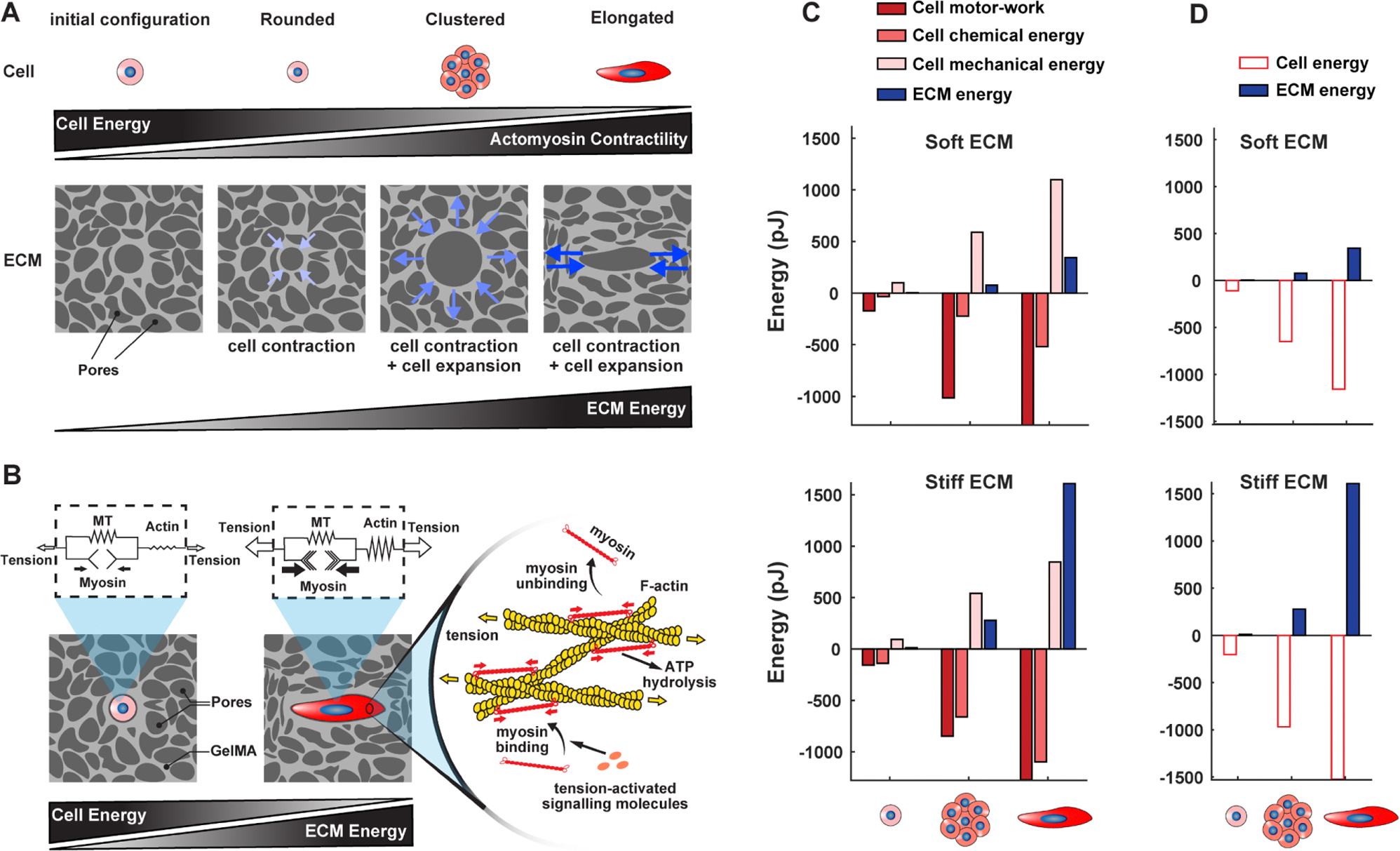
Energy-based model reveals distinct energy profiles for rounded, elongated, and clustered morphologies. (A-B) Cells initially exhibit a rounded morphology within the extracellular matrix (ECM). Over time, they may either maintain this rounded shape or transition into elongated or clustered morphologies. The model shows that the elongated morphology has the highest ECM mechanical energy because elongation requires substantial deformation of the surrounding ECM. However, elongated cells have the lowest cell energy due to elevated actomyosin contractility, which results from ATP hydrolysis and the associated release of chemical energy. This chemical energy release is the primary factor lowering the total cell energy in elongated cells. (C) Detailed breakdown of energy components: Cell energy was estimated as the sum of three key contributions: (i) chemical energy released during ATP hydrolysis as myosin motors bind to actin filaments, (ii) motor-work energy, representing the mechanical work generated by actomyosin contractility, and (iii) cell mechanical energy, which includes the passive strain energy stored in cytoskeletal structures and the external work performed by them. Each of these three energy components and the ECM mechanical energy were estimated and plotted. Positive energy values indicate resistance to adopting a given morphology, while negative values favor it. (D) The three cell energy components were summed to obtain the total cell energy, which was plotted alongside ECM mechanical energy. The results show that the elongated morphology has the lowest total cell energy but the highest ECM mechanical energy, while the rounded morphology has the lowest ECM mechanical energy but the highest cell energy. The clustered morphology lies between these two extremes.

To understand which morphological state (rounded, elongated, or clustered) a cell is most likely to adopt in the ECM, we used a theoretical model based on the minimum energy principle. Based on this principle, we hypothesized that a cell will take the shape that requires the least total energy. We implemented the model within a three-dimensional finite element framework to simulate cell spreading and contraction across three morphological states: rounded, elongated, and clustered. For each morphological state, we calculated the key energy components contributing to the total cellular energy in both soft and stiff extracellular matrices (Fig. 2A, S6). In this framework, a positive energy component indicates resistance to a particular morphology, with higher values representing greater opposition to that state. Conversely, a negative energy component favors a given morphology, with more negative values indicating a stronger preference for that state [39].

One major component of the total energy is ECM mechanical energy, which represents the energy a cell must expend to deform the ECM to take a particular morphology. Initially, cells are small and round, measuring ~ 20 μm in diameter, as shown in our experiments in Fig. S7. For a cell to transition into a mesenchymal-like morphology, it must spread and push against the surrounding ECM, elongating its long axis to roughly 100 μm, as observed experimentally in Fig. S7. This process requires mechanical work, making ECM mechanical energy an unfavorable factor for elongated morphology. However, for a rounded morphology, ECM mechanical energy remains negligible because the initial cell size (~ 20 μm) is equal to or smaller than the ECM pore size (Fig. 1C). This trend is schematically illustrated in Fig. 2A, where the rounded state has the lowest ECM mechanical energy, while the elongated state has the highest. Therefore, based solely on ECM mechanical energy, the rounded morphology should be the most favorable.

However, total energy also includes cell energy, which we estimate as the sum of three key components (Supplementary Information 2.1): (i) chemical energy, which is released when myosin motors bind to actin filaments, hydrolyzing ATP; (ii) motor-work energy, which represents the mechanical work performed by actomyosin contractility; and (iii) cell mechanical energy, which includes both the passive strain energy stored in cytoskeletal components (such as microtubules and actin filaments) and the external work performed by these structures. We estimated each of these three components separately (Fig. 2C) and then summed them to obtain the cell energy. This cell energy was plotted alongside the ECM energy in Fig. 2D.

These energy components vary depending on cell morphology, since different shapes require different levels of contractile machinery activation and cytoskeletal organization. We recently demonstrated that cell elongation generates strong anisotropic cytoskeletal tension, which in turn amplifies actomyosin contractility, leading to increased myosin phosphorylation and enhanced actin polymerization [39,55]. This, in turn, increases ATP hydrolysis and chemical energy release. As a result, the elongated morphology exhibits the highest actomyosin contractility in our simulations (Fig. 2A,B), yielding the most negative cell energy values (Fig. 2D). Conversely, the rounded morphology is associated with the lowest contractility and, therefore, the least negative cell energy. In summary, the elongated state has the lowest cell energy but the highest ECM mechanical energy, while the rounded state exhibits the opposite trend. The clustered state falls between these two extremes (Fig. 2A).

**Fig. 3.**
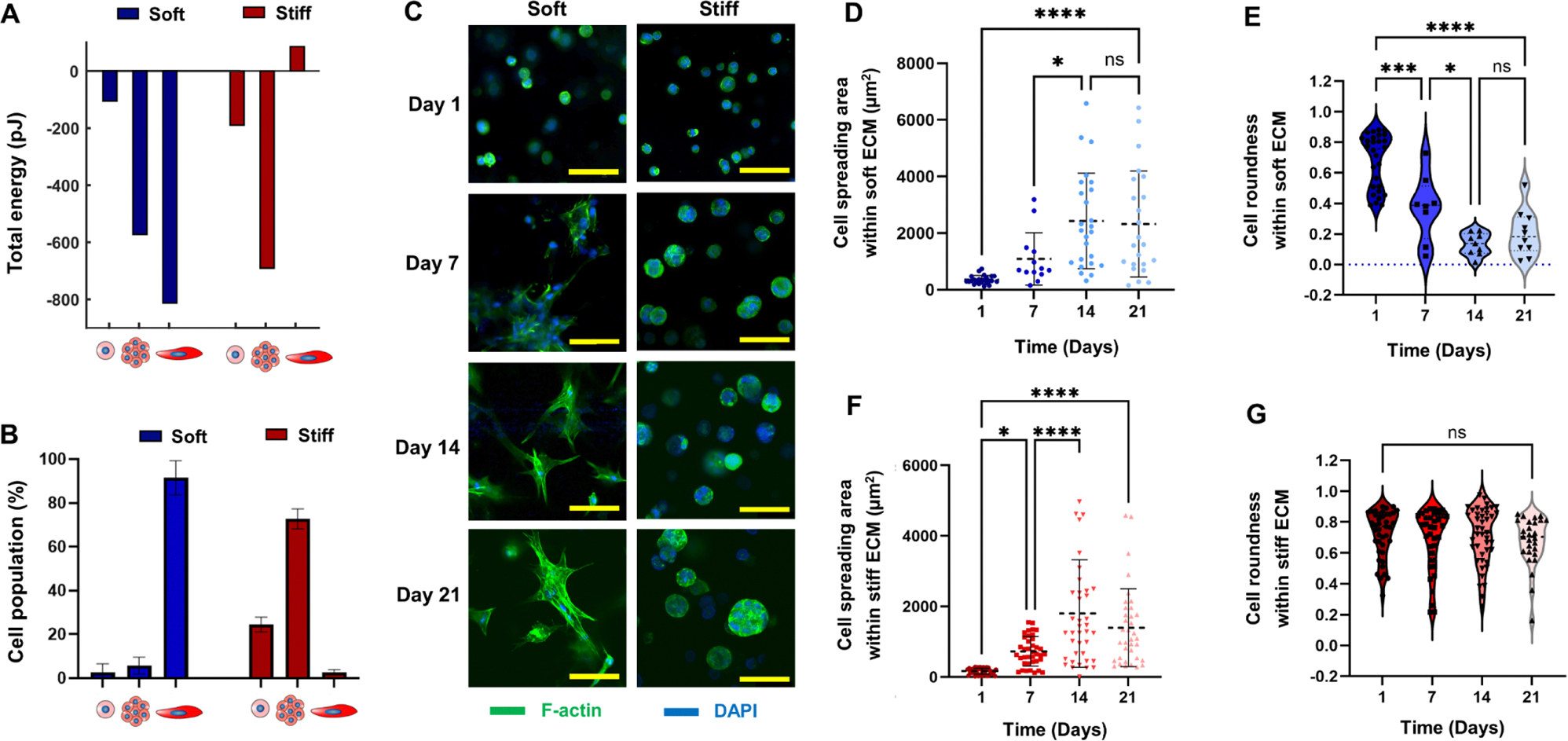
In agreement with our theoretical results, our experimental observations show that ECM stiffness dictates distinct morphological transitions. (A) Total energy for each morphological state was estimated by summing ECM mechanical energy and cell energy in both soft and stiff ECMs. The model shows that in soft ECM, the elongated morphology has the lowest total energy, making it the most favorable state, followed by clustered and rounded morphologies. In stiff ECM, the clustered morphology has the lowest total energy, followed by the rounded morphology, while the elongated morphology is the least favorable. (B) These theoretical results are consistent with our experimental measurements, where we quantified the percentage of cells adopting different morphological states (rounded, clustered, elongated) in soft and stiff ECMs (*n* ≥ 20 cells per condition). (C) Representative images illustrating morphological changes of cells within soft and stiff ECMs over 21 days, visualized by immunofluorescence staining of F-actin (green, phalloidin) and nuclei (blue, DAPI). (scale bar, 50 μm). Images are from three independent biological replicates, each containing three hydrogel technical replicates and triplicate fields of view per sample. (D-G) Quantification of cell area and shape evolution in soft and stiff ECMs over 21 days (*n* = 3 samples per condition with *N* ≥ 10 independent cells/clusters per sample). At day 1, cells exhibited similar rounded morphologies with low spreading areas in both ECM types. Over time, cells in soft ECM adopted an elongated morphology, as indicated by a gradual reduction in roundness, whereas cells in stiff ECM proliferated and formed clusters while maintaining a rounded shape. ns *p >* 0.05, *p ≤ 0.05, **p ≤ 0.01, ***p ≤ 0.001, ****p ≤ 0.0001. Bar heights represent mean values, and error bars represent ± standard error of the mean.

By summing the ECM mechanical energy and cell energy, we calculated the total energy for each morphological state in both soft and stiff ECMs (Fig. 3A). The model shows that in soft ECM, the elongated morphology has the lowest total energy, making it the most favorable state, followed by the clustered and rounded morphologies. In contrast, in stiff ECM, the clustered morphology has the lowest total energy, followed by the rounded morphology, while the elongated morphology is the least favorable. This shift emerges because, in soft ECM, the energy cost of spreading and elongating is relatively low due to the ECM’s low stiffness and large pore size (Fig. 2D). Subsequently, the cell can afford this mechanical cost in exchange for achieving high actomyosin contractility, which is energetically beneficial by releasing chemical energy. However, in stiff ECM, the energy required to deform the ECM is significantly higher, making elongation energetically prohibitive. As a result, clustered and rounded morphologies become more favorable in stiff ECM conditions (Fig. 3A).

### 3.3. Experiments: cell morphological transitions within soft and stiff ECMs

To examine the theoretical results, we cultured MDA-MB-231 cells within soft and stiff ECMs and monitored their morphological transitions over 21 days. Initially, at day 1, cells in both soft and stiff ECMs predominantly exhibited a rounded morphology (Fig. 3C). However, over time, distinct transitions emerged depending on ECM stiffness. In soft ECM, cells gradually transitioned from individual rounded morphologies to an elongated state, whereas in stiff ECM, they transitioned from a rounded morphology to a clustered state.

We quantified these morphological changes in Fig. 3B, which presents the percentage of cells in each state (rounded, elongated, and clustered) across different ECM conditions. Consistent with model results (Fig. 3A), elongated cells became the dominant population in soft ECM, while clustered cells were the most prevalent in stiff ECM.

Further quantitative analysis is shown in Fig. 3D–G, where we tracked the evolution of cell shape over time. At day 1, cells in both ECM conditions exhibited similar round morphologies with low spreading areas (Fig. 3D,F). However, in soft ECM, cells progressively adopted an elongated morphology over 21 days, as evidenced by a gradual reduction in roundness (Fig. 3E). In contrast, in stiff ECM, cells proliferated, forming clusters while maintaining their spherical shape, reflected by their consistent roundness over time (Fig. 3G). These experiments are in agreement with our theoretical results, demonstrating that ECM stiffness governs distinct morphological transitions: elongation in soft ECM and clustering in stiff ECM.

### 3.4. ECM mechanical properties, not chemical composition, govern cell morphological transitions

**Fig. 4.**
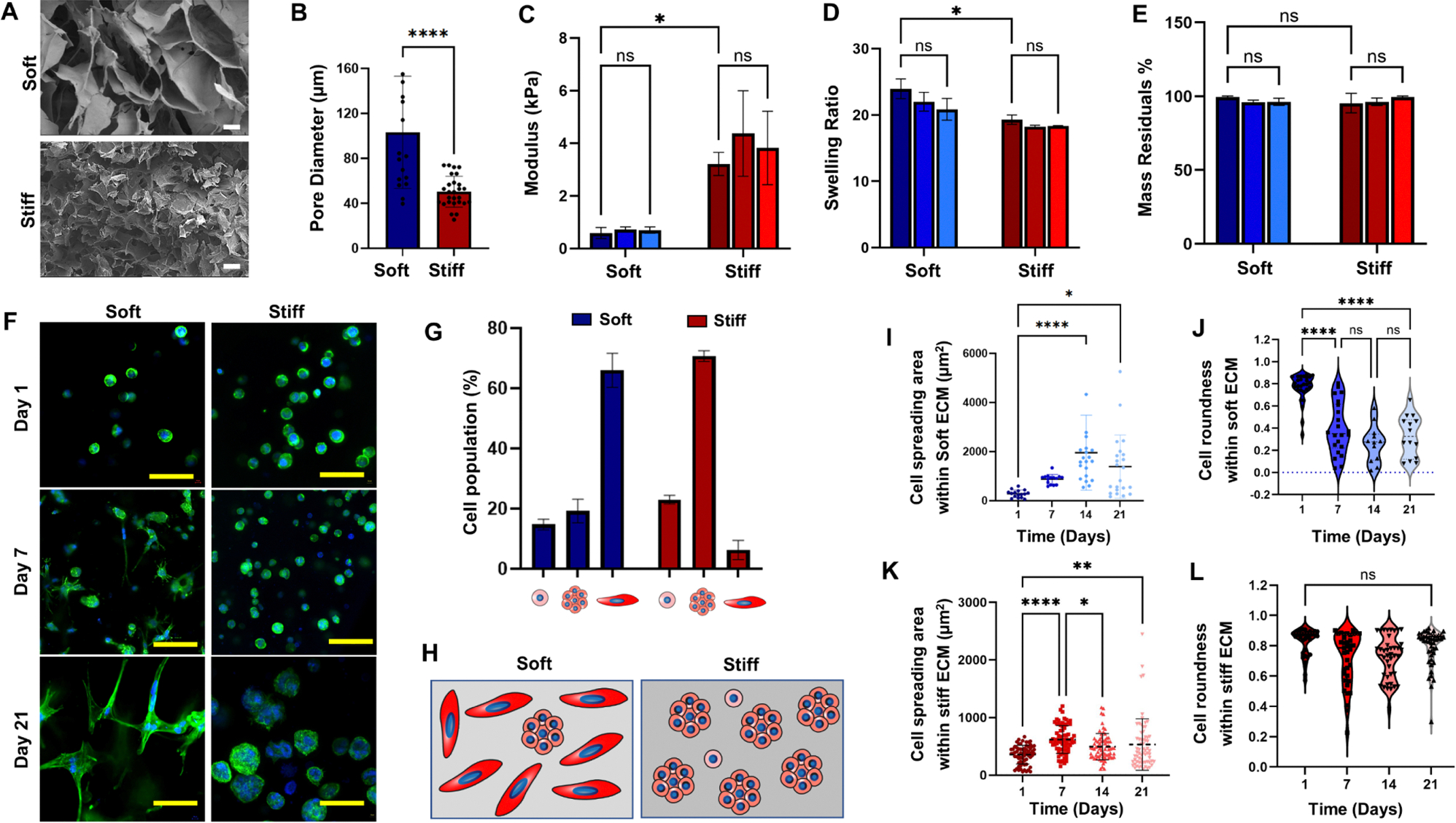
Composite ECMs replicate the mechanical regulation of cell morphology independent of chemical composition. (A) SEM images of soft and stiff composite HAMA-GelMA hydrogels (scale bar, 100 μm). (B) Quantification of pore size in soft and stiff composite hydrogels (*n* = 3 SEM images). (C-E) Characterization of mechanical and structural stability of composite hydrogels over time, including (C) compressive modulus, (D) swelling ratio, and (E) mass residual of cell-laden hydrogels measured at days 1, 7, and 21. (F) Representative immunofluorescence images of F-actin (green, phalloidin) and nuclei (blue, DAPI) showing cell morphology in composite hydrogels (scale bar, 50 μm). Images are from three independent biological replicates, each containing three hydrogel technical replicates and triplicate fields of view per sample. (G) Quantification of the percentage of cells adopting different morphological states (rounded, clustered, elongated) in soft and stiff composite hydrogels. (H) Schematic illustration of cell morphology within soft and stiff composite hydrogels. (I-L) Quantification of cell area and shape evolution in soft and stiff composite hydrogels over 21 days (*n* = 3 samples per condition with *N* ≥ 8 independent cells/clusters per sample). ns *p >* 0.05, *p ≤ 0.05, **p ≤ 0.01, ***p ≤ 0.001, ****p ≤ 0.0001. Bar heights represent mean values, and error bars represent ± standard error of the mean.

To determine whether the observed cell morphological transitions were dictated by ECM mechanical properties rather than chemical composition, we prepared two new ECMs composed of a hybrid HAMA-GelMA hydrogel, in contrast to the GelMA-only ECMs used previously. While these composite ECMs differed chemically from GelMA ECMs, our measurements confirmed that they maintained similar mechanical properties. Specifically, similar to the GelMA ECMs in Fig. 1, the composite ECMs exhibited distinct mechanical properties: a soft ECM (~ 1 kPa) with large pores and a stiff ECM (~ 3.5 kPa) with smaller pores (Fig. 4A–C, S8). Additionally, swelling ratio (Fig. 4D) and mass residual (Fig. 4E) measurements for the composite ECMs closely matched those of the GelMA ECMs (Fig. 1E,F). Notably, the mass residual remained unchanged, indicating minimal ECM degradation and confirming that cell-produced MMP did not significantly alter the ECM.

After verifying that the composite ECMs replicated the physical properties of the GelMA ECMs, we examined whether differences in chemical composition influenced cell morphology. If ECM chemistry played a significant role, we would expect differences in cell morphological transitions between composite and GelMA ECMs. However, our results showed that cell behavior remained consistent across both ECM types. Similar to the GelMA ECMs, at day 1, cells in both soft and stiff composite ECMs exhibited a rounded morphology (Fig. 4F). Over time, distinct transitions emerged depending on ECM stiffness, mirroring our previous results in Fig. 3. In the soft composite ECM, cells gradually transitioned from individual rounded morphologies to an elongated state, whereas in the stiff composite ECM, they shifted toward a clustered state (Fig. 4F).

Quantification of these morphological changes further confirmed the consistency of results between ECM types. As observed in GelMA ECMs, elongated cells became the dominant population in soft composite ECMs, while clustered cells were most prevalent in stiff composite ECMs (Fig. 4G,H). Tracking the evolution of cell shape over time showed that, at day 1, cells in both composite ECMs displayed rounded morphologies with low spreading areas (Fig. 4I,K). In soft composite ECMs, cells progressively adopted an elongated morphology over 21 days, as reflected by a gradual reduction in roundness (Fig. 4J). In contrast, cells in stiff composite ECMs proliferated, forming clusters while maintaining their spherical shape, as indicated by their consistent roundness over time (Fig. 4L). Overall, these findings reinforce our results from GelMA ECMs, as well as our theoretical results, demonstrating that ECM physical properties, rather than chemical composition, govern cell morphological transitions.

## 4. Discussion and conclusions

In this study, we combined theoretical modeling and experimental validation to uncover how ECM physical properties guide cancer cell morphology in three-dimensional microenvironments. By balancing the mechanical energy cost of ECM deformation against the energetic gains from actomyosin contractility, our model shows that cells adopt morphologies that minimize total system energy, consistent with previous chemo-mechanical modeling frameworks [36–39]. Importantly, the model’s prediction that cells in soft ECMs elongate because they benefit energetically from elevated actomyosin contractility is experimentally supported by our inhibitor studies (Fig. S9), where disruption of either actin polymerization (cytochalasin D) or myosin activity (blebbistatin) prevented cells in soft ECMs from transitioning to the elongated state. Together, our theoretical and experimental results demonstrate that ECM stiffness and pore architecture govern transitions between rounded, elongated, and clustered morphologies. These findings are in agreement with earlier mechanobiology studies, which reported stiffness-dependent invasion and contractility-mediated morphological regulation [16,20,24].

A key insight emerging from our work is that different morphological states likely represent distinct functional programs with important implications for tumor invasion. Cells that adopt an elongated morphology exhibit high levels of actomyosin contractility, a hallmark of migratory and invasive behavior. In contrast, cells that form clusters prioritize proliferation and cell-cell adhesion, promoting tumor expansion. Thus, ECM mechanics may determine cell shape and bias the balance between two fundamental cancer strategies: local tumor growth *versus* systemic dissemination through invasion [56].

This duality reveals an important principle: softer and more porous ECM environments may prime cancer cells toward an invasive, migratory phenotype, whereas stiffer and denser environments may favor clustering and tumor mass expansion. Given that patient tumors are mechanically heterogeneous, with regions of variable stiffness and porosity, this mechanical heterogeneity could locally program cancer cells to either grow in place or escape and invade surrounding tissues [20]. Future work should extend these principles to other cell types, including different cancer cells and organotypic models, and screen therapeutics to manipulate the mechanical landscape of tumors to design, control, and monitor cancer cell behavior.

While offering a controlled environment, our current experimental model possesses certain limitations. Specifically, it lacks crucial fibrous proteins, such as collagen fibrils, and common biophysical heterogeneity cues, including pore anisotropy and inhomogeneity, which are characteristic of complex ECM-derived hydrogels like Matrigel. Furthermore, the inherent fluid filtration within the culture system may influence dynamic cell-fluid interactions, potentially altering cellular responses compared to *in vivo* physiological conditions.

Another limitation of this study is that the effects of ECM viscoelasticity were not incorporated into our experimental design and theoretical model. In addition to stiffness and pore architecture, ECM viscoelasticity has emerged as a critical regulator of cellular behavior, particularly in the context of cancer progression. Unlike purely elastic matrices, viscoelastic materials dissipate stress over time, enabling cells to remodel their surroundings, reorganize their cytoskeleton, and adapt their morphology in different ways. Recent studies have shown that viscoelastic relaxation of the ECM can facilitate cell spreading, protrusion formation, and migration by lowering long-term mechanical resistance [57,58]. Similarly, viscoelastic hydrogels have been shown to accelerate cancer cell invasion, enhance stemness, and modulate mechanotransduction pathways that control transcriptional programs associated with metastasis [59–61]. The full mechanical landscape sensed by a cell includes not only stiffness and confinement, but also the rate at which the ECM relaxes under cell-generated forces. In addition to ECM viscoelasticity, the viscoelasticity of cellular components also plays a major role in determining how cells generate forces, reorganize their actin networks, and transition between various morphological states. The recent works highlight that cells themselves behave as time-dependent materials, [62–64] with contractility, actin polymerization, and microtubule dynamics all exhibiting characteristic relaxation times. These internal viscoelastic properties influence how cells respond to extracellular mechanical cues and can enable morphological transitions even in matrices with identical stiffness. Taken together, integrating viscoelasticity into future modeling and experiments would provide an even more comprehensive picture of how the full mechanical spectrum of the tumor microenvironment (elasticity, viscoelasticity, and microarchitecture) governs cancer cell morphology and fate.

In summary, our results reveal how ECM physical properties dictate cancer cell morphology in 3D and provide a mechanistic basis for understanding morphological heterogeneity within tumors. These insights lay the foundation for future efforts to predict and control tumor cell behavior using engineered microenvironments, with potential applications in metastasis modeling, drug screening, and the design of biomaterials that modulate cancer progression.

## Supplementary Material

SI

Supplementary material associated with this article can be found, in the online version, at doi:10.1016/j.actbio.2025.12.008.
